# Fibroblast growth factor receptor signaling in estrogen receptor-positive breast cancer: mechanisms and role in endocrine resistance

**DOI:** 10.3389/fonc.2024.1406951

**Published:** 2024-07-08

**Authors:** Arnaldo Marin, Fernanda Morales, Benjamín Walbaum

**Affiliations:** ^1^ Doctoral Program in Medical Sciences, Faculty of Medicine, University of Chile, Santiago, Chile; ^2^ Oncology Program, Faculty of Medicine, University of Chile, Santiago, Chile; ^3^ Department of Basic and Clinical Oncology, Faculty of Medicine, University of Chile, Santiago, Chile; ^4^ Medical Oncology Department, Hospital Clinic, Barcelona, Spain; ^5^ August Pi i Sunyer Biomedical Research Institute (IDIBAPS), Barcelona, Spain; ^6^ Department of Hematology-Oncology, School of Medicine, Pontificia Universidad Católica de Chile, Santiago, Chile

**Keywords:** endocrine resistance, FGFR signaling, breast cancer, ER positive, TKI - tyrosine kinase inhibitor

## Abstract

Fibroblast Growth Factor Receptors (FGFRs) play a significant role in Estrogen Receptor-positive (ER+) breast cancer by contributing to tumorigenesis and endocrine resistance. This review explores the structure, signaling pathways, and implications of FGFRs, particularly FGFR1, FGFR2, FGFR3, and FGFR4, in ER+ breast cancer. FGFR1 is frequently amplified, especially in aggressive Luminal B-like tumors, and its amplification is associated with poor prognosis and treatment resistance. The co-amplification of FGFR1 with oncogenes like EIF4EBP1 and NSD3 complicates its role as a standalone oncogenic driver. FGFR2 amplification, though less common, is critical in hormone receptor regulation, driving proliferation and treatment resistance. FGFR3 and FGFR4 also contribute to endocrine resistance through various mechanisms, including the activation of alternate signaling pathways like PI3K/AKT/mTOR and RAS/RAF/MEK/ERK. Endocrine resistance remains a major clinical challenge, with around 70% of breast cancers initially hormone receptor positive. Despite the success of CDK 4/6 inhibitors in combination with endocrine therapy (ET), resistance often develops, necessitating new treatment strategies. FGFR inhibitors have shown potential in preclinical studies, but clinical trials have yielded limited success due to off-target toxicities and lack of predictive biomarkers. Current clinical trials, including those evaluating FGFR inhibitors like erdafitinib, lucitanib, and dovitinib, have demonstrated mixed outcomes, underscoring the complexity of FGFR signaling in breast cancer. The interplay between FGFR and other signaling pathways highlights the need for comprehensive molecular profiling and personalized treatment approaches. Future research should focus on identifying robust biomarkers and developing combination therapies to enhance the efficacy of FGFR-targeted treatments. In conclusion, targeting FGFR signaling in ER+ breast cancer presents both challenges and opportunities. A deeper understanding of the molecular mechanisms and resistance pathways is crucial for the successful integration of FGFR inhibitors into clinical practice, aiming to improve outcomes for patients with endocrine-resistant breast cancer.

## Introduction

The human fibroblast growth factor receptor (FGFR) family comprises FGFR1, FGFR2, FGFR3, and FGFR4. These highly conserved receptor tyrosine kinases (RTKs) are in the cell membrane (1, 2). Additionally, FGFR5 (also known as fibroblast growth factor receptor-like 1; FGFRL1), which lacks the intracellular kinase domain, is classified as the fifth member of the FGFR family (3). FGFRs are activated through the interaction of fibroblast growth factor (FGF) ligands. The mammalian FGF family comprises 22 members that are classified into 6 subfamilies by their mechanisms of action. In paracrine signaling (canonical FGFs) there are 5 subfamilies: FGF1 (FGF1 and FGF2), FGF4 (FGF4, FGF5, and FGF6), FGF7 (FGF3, FGF7, FGF10, and FGF22), FGF8 (FGF8, FGF17, and FGF18), and FGF9 (FGF9, FGF16, and FGF20). Furthermore, the endocrine subfamily consists of FGF19 (FGF19, FGF21, and FGF23) (4–6). FGF15 corresponds to the mouse ortholog of human FGF19 (FGF15/FGF19) (7, 8). In paracrine cellular communication, FGFs participate in various cellular processes that include cell proliferation, migration, differentiation and cell survival (9, 10), while endocrine secreted FGFs are released into the blood and can reach different tissues of the body, participating in the regulation of the bile acids, carbohydrates, lipids and phosphate/vitamin D metabolism (11–19).

## FGFR structure

**Figure 1:**
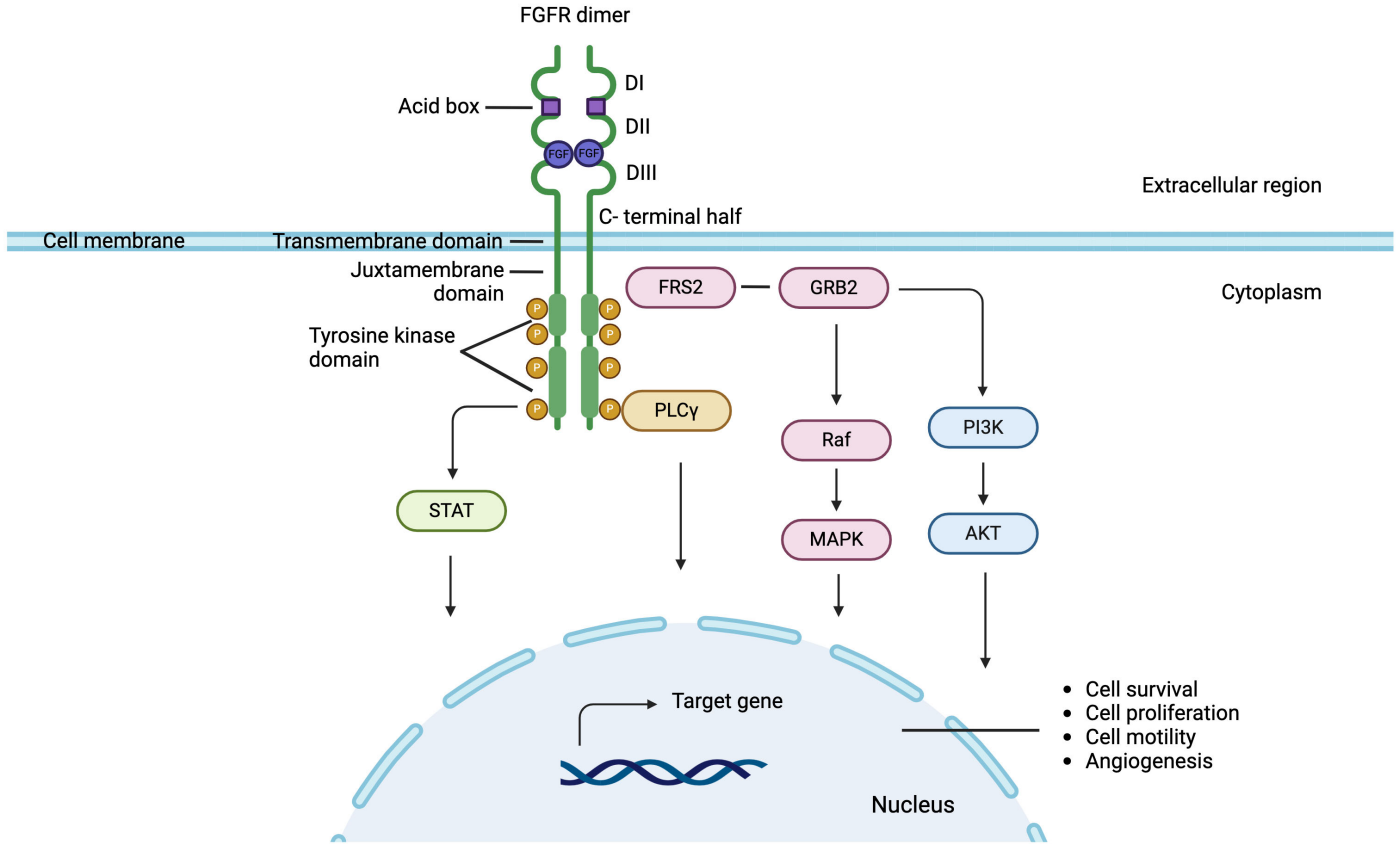
FGFR signaling pathway in breast cancer: FGF binds to the extracellular domain of FGFR leading to receptor dimerization and through phosphorylation inducing downstream activation of canonical pathways. Signaling pathways dependent of FGF-FGFR axis, include Fibroblast Growth Factor Receptor Substrate 2 (FRS2) Ras-mitogen-activated protein kinase (MAPK), phosphatidylinositol-3 kinase (PI3K), protein kinase B (AKT), phospholipase Cγ1 (PLCγ1) and signal transducer and activator of transcription (STAT).

The structure of FGFR1 – FGFR4 from N- to C-terminus contains an extracellular region comprising three immunoglobulin-like loop domains (Ig domains; DI, DII and DII) (5, 20). Between DI and DII domains is the acid box, a conserved motif rich in aspartic acid residues (21). This is followed by a transmembrane region formed by a single α-helix, a juxtamembrane domain, and two intracellular tyrosine kinase domains (1, 5, 20, 22) FGFR5 has three extracellular domains similar to immunoglobulins, a transmembrane domain and a short intracellular tail without a tyrosine kinase domain that presents a histidine-rich region (23, 24) (**Figure 1**).

## Overview of FGF/FGFR signaling pathways

FGFs are molecules that bind to FGFR monomers in their extracellular region between domains II and III (20, 25). Ligand binding induces conformational changes and dimerization of FGFR, which trigger transphosphorylation within the dimerized receptor pair, and therefore the activation of cytosolic kinase domains (20, 26–30). The autophosphorylated domains of FGFRs serve as specific binding sites for different substrates, which in turn are phosphorylated by active receptors (23, 31). Through this mechanism, various downstream effector molecules are activated and participate in the signaling pathways of the FGF-FGFR axis, including Fibroblast Growth Factor Receptor Substrate 2 (FRS2) Ras-mitogen-activated protein kinase (MAPK), phosphatidylinositol-3 kinase (PI3K), protein kinase B (AKT), phospholipase Cγ1 (PLCγ1) and signal transducer and activator of transcription (STAT). FRS2, a protein located in the plasma membrane, is the main substrate of FGFR. Following activation by phosphorylation, FRS2 binds and recruits the adapter protein Growth factor receptor-bound protein 2 (GRB2) triggering the activation of the Ras-MAPK signaling pathway, which regulates the mitogenic cellular response, cell cycle regulation and cell survival (32–37). Also activated FRS2 results in the phosphorylation and recruitment of PI3K, activating the PI3K-AKT signaling cascade involved in cell survival and angiogénesis (38–44). Furthermore, tyrosine residues of FGFR are also recognized by PLCγ1 and STATs, proteins related to cell motility and cell transformation, respectively (40, 45–48) (**Figure 1**).

## FGFR in estrogen receptor-positive breast cancer

Estrogen receptor-positive (ER^+^) tumors represent the most frequent subtype in breast cancer (BC) across all ages (49) yet unveiling its biology, beyond the estrogenic receptor pathway, has been challenging. Several genomic alterations in both FGFs and FGFRs genes have been reported in BC patients, which vary in their presentation according to BC subtypes and stage (50–52). Among ER^+^ BC, FGFR signaling pathway has emerged as a relevant factor in tumorigenesis and treatment resistance (53, 54). FGFR1 gene alterations are the most frequently detected, mainly characterized by the amplification of 8p11–12 locus, present in 10 -15% patients and reaching over 16% to 27% in aggressive tumors (Luminal B-like) (55). FGFR1 signaling appears to play a central role in proliferation, migration, and survival of mammary cells and its amplification is a relevant prognostic factor as it has shown to strongly predict poorer overall survival among BC patients (56). However, FGFR1 amplification generally occurs alongside the co-amplification of several neighboring genes, of which EIF4EBP1 and NSD3 have been identified as oncogenic, the former with a proven oncogenic role in luminal BCs (57, 58). Thus, FGFR1 is not the only oncogene present in the 8p11.23 amplicon, which challenges its role as an oncogenic driver and the real dependency or addiction to FGFR1 signaling (59). Moreover, there is a low concordance between FGFR1 amplification and final protein expression (60). While, simultaneously, FGFR1 overexpression can occur independently of gene amplification, as proven in lung cancer studies (61).

Other FGFR alterations have been implicated in ER^+^ breast cancer. Though infrequent (around 3%) and mainly found in ER-negative BC, FGFR2 amplification plays a predominant role as a driver oncogene. The FGFR2 amplicon is shorter, compared to FGFR1, and includes fewer coamplified genes, thus being more centered on FGFR2, better predicting its oncogenic role. FGFR2 regulates FGFR3 expression, leading to high levels of FGFR3 in FGFR2-activated tumors. Additionally, the origin of FGF2 and its interaction with FGFR2, may determine the cell line context. Either autocrine, with high FGF2 expression seen in basal-like breast cancer cell lines, or paracrine, where it is stromal FGF2 that activates hormone receptors and induces proliferation of luminal cancer cells. Consequently, preclinical studies have shown that FGFR2-amplified cell lines are more sensitive to inhibition compared to those with FGFR1 amplification (62–64).

Furthermore, FGFR4 gene amplification is detected in around 10% of patients and its overexpression tends to be found more frequently in HER2 enriched tumors (65). As opposed to other histologies, point mutations, fusions, nuclear FGFR expression and FGF ligand amplification are initially infrequent in BC, however their clonal acquisition after treatment does play a role in ER^+^ BC, especially after treatment exposure with endocrine therapy (ET) and Cyclin–dependent kinase 4/6 inhibitors (CDK 4/6 inhibitors) (66, 67).

## FGFR and ET resistance

Around 70% of BC are initially classified as hormone receptor-positive (68), meaning that the estrogen receptor signaling pathway is the main driver of cancer cell growth and tumor survival. Whereby endocrine therapy is the mainstay of its treatment both in early BC and in advanced BC (ABC) (69). Among patients with distant recurrence, however, around 14% of patients are primarily resistant to estrogen deprivation therapy, and the vast majority acquire resistance at some point of their treatment (70, 71). This has led to the development of targeted therapies such as CDK 4/6 inhibitors that in combination with ET have dramatically changed the natural history of this disease, with median progression free survival for first line treatment of ET plus CDK 4/6 inhibitors reaching over 24 months (72). Still, most patients eventually progress due to drug resistance, prompting for changes in the treatment strategy. Yet, further treatment lines are less effective, without one defined subsequent therapy (73). Resistant mechanisms have been extensively studied, and different treatments have been developed to overcome resistance. Estrogen receptor 1 (ESR1) mutation detection, identified as a strong predictor of standard ET resistance, has led to the approval of elacestrant, an oral selective estrogen receptor degrader (SERD) in patients progressing after ET in combination with CDK 4/6 inhibitors (74). Whereas PIK3CA gene mutations, truncal mutations present at initial stages in over 30% of patients, have shown to predict worst response to ET (75), and led to the development of PI3K inhibitors such as alpelisib (76) and the recently presented inavolisib (77). The latter showing interesting results among patients defined as endocrine resistant, due to early relapse con ET. Moreover, inhibitors of the AKT pathway have demonstrated to play a relevant role in ET sequence strategy when either PIK3CA, AKT1 or PTEN mutations are detected (78). This led to the Food and Drug Administration (FDA) approval of capivasertib for ER^+^/HER2^-^ advanced BC patients following progression on at least one endocrine-based regimen (79). Loss of ER and other alterations, such as mutations in RAS/RAF/MEK pathway, have also been described as relevant resistance mechanisms favoring non estrogen dependent pathways, however drug development targeting this pathway has been elusive in BC (80).

FGFR1 gene amplification has an often-unaccounted role in endocrine resistance. Several resistance mechanisms have been described, including the activation of PI3K/AKT/mTOR pathway, persistent RAS/RAF/MEK/ERK pathway activation and cyclin D1 overexpression (81, 82). Upon inhibition of CDK4/6, AKT signaling is upregulated, which contribute to the accumulation of cyclin D1. This accumulation allows cyclin D1 to associate with CDK2, bypassing CDK4/6 inhibition, and promoting S phase progression. Notably, up to one-third of FGFR1-amplified tumors also harbor amplification of CCND1 gene, which encodes for cyclin D1 protein. Still, FGFR1 signaling promotes cell proliferation by directly upregulating the CCND1 gene, with higher levels of cyclin D1 mRNA observed even among FGFR1-amplified tumors without CCND1 amplification (83). Additionally, FGFR1 induces ET plus CDK 4/6-inhibition resistance by activating cancer cell stemness [(84) 21098263] and by upregulating Wnt/β-catenin signaling (85). ctDNA samples collected after progression on CDK 4/6-inhibitors showed an enrichment of FGFR pathway alterations, while FGFR1 amplifications detected in baseline ctDNA samples of patients included in Monaleesa -2 trial was associated with worse survival outcomes (83).

This has led to a growing body of research exploring the use of FGFR inhibitors, with preclinical data suggesting that FGFR1 amplified BC could maintain ERα pathway activated even with estrogen deprivation therapy (86). Amplification of the chromosomal region 8p11-12, the genomic location of FGFR1, is the most frequent FGFR alteration in BC, furthermore, Formisano et al. (86), measured FGFR1 prior and post preoperative ET and confirmed an increase in FGFR1 levels in post-treatment samples. Suggesting that FGFR1 amplification represents not only an intrinsic resistant mechanism, but also an adaptive mechanism of escape to antiestrogen treatment. Preclinical data have also shown that tumor cells that harbor FGFR1 amplification when treated with ET in combinations with CDK 4/6 inhibitors reach significantly lower levels of cell cycle arrest state (83, 87), with reduced cell senescence (measured by senescence-associated (SA) β-galactosidase-positive cells) (88). Ever more relevant considering the role of senescent cells as elimination route of tumor cells when treated with cytostatics therapies (89). The addition of anti FGFR1 drugs could reverse this condition and has led to an important number of clinical trials evaluating its effectiveness and safety. However, as stated one third of these tumors present other co-occurring pathway altered functions and in up to over 20% of patients there is discordance between FGFR1 amplification and overexpression (81), underscoring the need to better define and quantify FGFR amplification.

Regarding FGFR2, several reports have shown a tight interaction between progesterone receptor (PR), FGFR2 and STAT5 proteins. Wherein stromal FGF2 and progesterone exert overlapping functions by activating common signaling pathways including ERK, AKT and STAT5. Thereby enabling the formation of a nuclear complex between PR, FGFR2 and STAT5. These complexes lead to the expression of PR/STAT-5 regulated proteins, favoring mammary gland differentiation and hormone-mediated growth. This way, among patients under endocrine suppression therapy, FGFR2-altered tumors may become independent of hormonal stimulation by switching to FGF2-mediated STAT5-dependent mechanisms for continuous cell proliferation (90). However, whole exome sequencing analysis of BC patients confirmed that both FGFR2 overexpression and mutations are rarely truncal mutations but acquired in metastatic biopsy after treatment progression (91).

Other FGFR alterations have been shown to promote endocrine resistance, including co-amplification of FGF 3/4/19 and CCND1, nuclear FGFR1 expression, FGFR3 overexpression, FGFR4 overexpression and FGFR4 mutations. However, their clinical relevance has yet to be determined (66, 92–94).

## Clinical evidence and ongoing trials

Various agents ranging from pan-FGFR inhibitors and specific FGFR 1, 2 and 3 inhibitors have been developed. With several FGFR inhibitors currently under development. So far for the treatment of solid tumor patients, erdafitinib for urothelial carcinoma (FGFR 2/3 alterations) (95), and futibatinib, infigratinib and pemigatinib for cholangiocarcinoma (FGFR2 fusions or rearrangements) have been approved (96, 97). While in BC, ongoing clinical trials and preclinical data have long been evaluating possible treatment strategies to block FGFR pathway by using small molecules tyrosine kinase inhibitors (TKIs). Notwithstanding, limited benefit has so far been the norm. Several molecules have been tested in different settings. Soria et al. showed promising results in 12 patients with advanced BC with FGFR1 amplified tumor treated with first generation multikinase inhibitor lucitanib (98). Later, in the phase II FINESSE study an exploratory biomarker analysis suggested a higher overall response rate in patients with FGFR1 amplifications defined by IHC H-score ≥50. However, treatment resulted in significant toxicities limiting optimal inhibitory dose (99). More recently, a phase Ib trial including 23 patients with ER^+^/HER2^-^ FGFR1 amplified metastatic BC previously exposed to CDK 4/6-inhibitors, combined erdafitinib with fulvestrant and palbociclib and showed a median PFS of only 3 months and a clinical benefit rate (CBR: complete response, partial response, or stable disease) of 28% at 6 months (100). While the phase II study EAY131 led by Gong and colleagues, which enrolled 18 patients with tumor FGFR 1-4 amplification to receive erdafitinib and included 9 patients with BC did not meet its primary endpoint, with only one patient with FGFR1-amplified BC showing prolonged survival (101). Additionally, the phase IIA RADICAL trial (102) which included 52 patients, compared the combination of anastrozole or letrozole to fexagratinib (AZD4547), a strong FGFR inhibitor (FGFR-1, 2 and 3), in endocrine resistant BC. Overall, objective response was only 10% (5/50) with a clinical benefit rate (CBR) after 28 weeks of therapy that reached 25%, with almost 27% (14) of patients discontinuing treatment due to adverse events, mainly ocular complications such as corneals ulcers, mucositis, elevated liver enzymes, anemia and fatigue. Two phase II trials have studied the benefit of the Dovitinib, a small molecule multikinase inhibitor of FGFR1, FGFR2, FGFR3 and other receptor tyrosine kinases. Preclinical data showed activity in FGFR positive and amplified BC xenograft models, reversing aromatase inhibitor resistance. Initially, single-agent Dovitinib showed activity in ER^+^/HER2^-^ FGFR1 amplified BC patients, particularly among patients with higher levels of amplification. However, CBR reached a meager 25% (103). Following these results, the combination with fulvestrant was tested in a double blind, phase II trial, however the study had to be stopped early due to slow accrual. Still, the final analysis, once again showed limited benefit, with no progression free survival difference in the intention to treat analysis, but with a significant difference (according to definition of study protocol) among patients from the predefined subgroup with FGF pathway amplification; reaching a median PFS of 10.9 months vs 5.5 months with fulvestrant as monotherapy and a response rate (ORR: Complete response or partial response) of 27.7% (104). Ongoing trials including a phase II trial evaluating futibatinib plus fulvestrant (NCT04024436) and the phase I dose escalation trial (ROGABREAST) with rogaratinib in combination with palbociclib and fulvestrant (NCT04483505), are yet to show any benefit. **Table 1** summarizes completed phase II or III randomized control trials.

**Table 1:** Phase II/III trials evaluating anti FGFR treatments in breast cancer patients.

Study Name	Drug	Target	FGFR inhibition site	Year	N°	Study Arms	Outcomes
FINESSE trial (Phase II) (99)	Lucitanib	VEGFR1-3, FGFR1-3, and PDGFRα/β	First-generation FGFR inhibitors Multi-TKIs ATP binding site of tyrosine kinase domain (99, 105–107)	2020	76	Cohort 1: FGFR1 amplified Cohort 2: FGFR1 nonamplified, 11q13 amplified Cohort 3: FGFR1 and 11q13 nonamplified	Cohort 1: ORR 19% (9% - 35%)
RADICAL Trial (Phase IIa) (102)	Fexagratinib (AZD4547)	FGFR1-3	Second-generation FGFR inhibitors Pan-FGFR inhibitor Reversible ATP binding site of tyrosine kinase domain (1, 102, 105–107)	2022	52	1 arm: AZD4547 (FGFR inhibitor) endocrine-resistant BC	ORR 10% and CBR 25%
NCI-MATCH ECOG-ACRIN Trial (EAY131) Subprotocol K1 (Phase II) (101)	Erdafitinib	FGFR1-4	Second-generation FGFR inhibitors Pan-FGFR inhibitor Reversible ATP binding site of tyrosine kinase domain (1, 101, 106, 107)	2024	18 (9*)	1 arm: Erdafitinib for FGFR1-4 amplified tumors	ORR 0%, CBR 22% (2/9 BC patients)
Dovitinib and Fulvestrant (Phase II) (104)	Dovitinib (TKI258)	FGFR1-4, VEGFR 1-3 & PDGFR α/β	First-generation FGFR inhibitors Multi-TKIs ATP binding site of tyrosine kinase domain (103–107)	2017	97	Fulvestrant plus Dovitinib or Placebo in postmenopausal patients with endocrine-resistant BC	mPFS: 5.5 (3.8-14.0) vs 5.5 (2.5-10.7) months (HR 0.68 0.41 - 1.14) *FGF pathway amplification:* mPFS 10.9 (3.5 -16.5) vs 5.5 (3.5 -16-4) months. HR: 0.64 (0.22 -18.86) mOS: NR (18.6 - NR) vs 25.9 (18.4 - NR) HR 0.81 (0.39-1.85) ORR 27.7% (15.6% - 42.6%) vs 10% (3.3% - 21.8%)
TKI258 trial (Phase II) (103)	Dovitinib (TKI258)			2013	81	1 arm: Dovitinib monotherapy	CBR 25%

FGFR, Fibroblast growth factor receptor; FGF, Fibroblast growth factor; VEGFR, Vascular endothelial growth factor receptor; PDGFR, Platelet derived growth factor receptor; BC, breast cancer; Multi-TKIs, multi-target tyrosine kinase inhibitors; ATP, adenosine triphosphate; mPFS, median progression free survival; mOS, median overall survival; ORR, overall response rate; HR, Hazard Ratio; NR, Not reached; CBR, clinical benefit rate (Complete response; partial response or stable disease).

*Patients with breast cancer.

Finally, new strategies are being evaluated to better tailor therapy with FGFR inhibitors by molecularly selecting BC patients that will benefit from treatment. As such, translational research evaluating FGFR1-4 mRNA levels has shown to be a better predictive biomarker (108). Future prospective trials including molecular determinants will help better profile patients with actionable FGFR alterations that will benefit from target therapy.

## Future directions and conclusions

The FGFR pathway represents an elusive target in the landscape of BC therapy. Particularly in ER^+^/HER2^-^ BC patients, where resistance to therapy remains a significant clinical challenge. Genomic alterations and dysregulation within the FGFR signaling axis, including FGFR amplifications, fusions and point mutations, have been associated as strong prognostic factors in BC, mainly as intrinsic or acquired resistant mechanisms to endocrine therapies, favoring disease progression and poorer survival outcomes for patients. Despite promising preclinical data evaluating FGFR as a relevant resistant pathway, ongoing clinical trials testing FGFR inhibitors have not been able to translate these findings into meaningful clinical benefits. Challenges such as lack of predictive biomarkers, due to the intricacies of signaling pathways in ER^+^/HER2^-^ BC, and significant adverse events due to off-target toxicities, have hindered the success of FGFR-targeted therapies in BC (109). Tumor heterogeneity mandates a deeper understanding of tumor biology including genomics analysis and new molecular technologies ranging from transcriptomics to RNA sequencing. Which may help better understand the complex interplay between FGFR signaling and other pathways involved in endocrine resistance (110). Furthermore, exploring innovative drug combinations could enable innovative approaches to overcome resistance mechanisms. Research efforts should focus on elucidating mechanisms of resistance, refining identification of predictive biomarkers, and exploring rational combination therapies to maximize efficacy while minimizing toxicity. Ultimately, the successful integration of FGFR-targeted agents into the clinical management of ER^+^/HER2^-^ BC should be focused on further improving patient outcomes by overcoming the limitations posed by endocrine resistance.

## Author contributions

AM: Conceptualization, Data curation, Formal analysis, Funding acquisition, Investigation, Methodology, Project administration, Resources, Software, Supervision, Validation, Visualization, Writing – original draft, Writing – review & editing. FM: Resources, Visualization, Writing – original draft. BW: Conceptualization, Investigation, Resources, Writing – original draft, Writing – review & editing.
